# A pre-market, open-label, single-arm study to evaluate b.Bone for posterolateral fusion treatment of degenerative thoracolumbar, lumbar or lumbosacral spinal disease (b.Spine clinical trial)

**DOI:** 10.3389/fbioe.2026.1851820

**Published:** 2026-09-25

**Authors:** Giuseppe Tedesco, Cristiana Griffoni, Jӧrg Franke, Giuliana Nervuti, Paolo Spinnato, Gabriele Bilancia, Zoe Berfelde, Katharina Franke, Riccardo Ghermandi, Valerio Pipola, Sonia Granados, Susanna Salvagno, Alessandro Gasbarrini, Giovanni Barbanti Brodano

**Affiliations:** 1 Complex Spinal Surgery Unit, IRCCS Istituto Ortopedico Rizzoli, Bologna, Italy; 2 Klinik für Orthopädie, Klinikum Magdeburg, Magdeburg, Germany; 3 Diagnostic and Interventional Radiology, IRCCS Istituto Ortopedico Rizzoli, Bologna, Italy; 4 GreenBone Ortho SpA, Faenza, Italy; 5 Department of Biomedical and Neuromotor Sciences, University of Bologna, Bologna, Italy

**Keywords:** adverse events, bone graft substitute, degenerative spine disease, posterolateral spinal fusion, spinal surgery

## Abstract

**Background:**

Spinal fusion requires a graft between the affected vertebrae to facilitate new bone ingrowth and subsequent fusion. While the gold standard for bone grafting remains the iliac crest autograft and local autograft, several different bone graft substitutes have been investigated. b.Bone is a EU class III Medical Device. b.Bone is a biomorphic calcium phosphate scaffold characterized by a hierarchical interconnected porous architecture designed to mimic the structural organization of trabecular bone. Although b.Bone is a CE-marked device to fill voids or gaps in the extremities and pelvis, this clinical investigation was specifically designed as a feasibility study to assess the safety and preliminary performance of b.Bone granules when used outside their intended scope.

**Methods:**

In this single-arm pilot study b.Bone was used as a bone graft expander mixed with autograft in a 1:1 ratio in posterolateral fusion surgery at two European clinical centers for the treatment of degenerative conditions of the thoracolumbar, lumbar or lumbosacral spine. Patients were treated with b.Bone up to three levels. Spinal fusion was assessed at 6 and 12 months follow up by CT scan. Moreover, surgical complications and quality of life were assessed.

**Results:**

15 patients (mean age 57.1 years, eight male and seven female) were enrolled. At 6 months successful radiographic fusion was achieved in 10 out of 15 patients (66.7% success rate). All cases achieved successful radiographic fusion at the treated spinal levels with b.Bone within 12 months post-surgery. Significant improvement in back pain was observed starting from 6 weeks post-surgery. Patients’ functional ability remained stable over time. During the clinical trial, a total of 27 adverse events (AEs) were observed. However, all these AEs were not related to the device.

**Conclusion:**

The results of this feasibility study support the non-inferiority of b.Bone as bone graft extender with respect to other synthetic ceramic biomaterials and reinforce the device’s benefit–risk profile in thoracolumbar instrumented fusion.

## 1 Introduction

Painful conditions of the spine are commonly caused or influenced by degenerative diseases, which include spinal stenosis, spondylolisthesis, and disc herniation (Barrey et al., 2019). Incidences of degenerative disc disease and spondylolisthesis have been gradually increasing in the society (Alfieri et al., 2013). Unbalanced loading and neural structure compression in these patients result in mechanical and neurological symptoms. Some patients’ condition and symptoms may be controlled with conservative therapies. When conservative treatments inadequately relieve pain or in the case of deteriorated neurological function, surgery may be considered to decompress the neural structures and stabilize the spine (Deyo et al., 2004; Høy et al., 2013). Lumbar spinal fusion is a widespread technique for the surgical management of degenerative lumbar pathology. Surgical options for lumbar spinal fusion include posterolateral fusion (PLF), anterior lumbar interbody fusion (ALIF), posterior lumbar interbody fusion (PLIF), or transforaminal lumbar interbody fusion (TLIF) (Mobbs et al., 2015). The thoracic spine is usually involved in fusion surgeries for scoliosis or similar deformities (Amara et al., 2019).

Spinal fusion surgery requires a graft between the affected vertebrae to facilitate new bone ingrowth and subsequent fusion (Morris et al., 2018). More than 2.2 million bone graft procedures are performed each year worldwide, including one million procedures in Europe, making bone the second most common transplanted tissue after blood (Zhang et al., 2025).

The gold standard for bone grafting in spinal surgery remains the iliac crest autograft and local autograft (spinous processes, laminae), harvested from either a donor site or the surgical site. Autologous bone possesses the three essential properties of osteogenesis, osteoinductivity, and osteoconductivity, all of which are crucial for optimizing spine arthrodesis (Tavares et al., 2022). However, the availability and quality of autologous bone may be limited, contingent upon factors such as patient age and biology. Moreover, the use of iliac crest bone graft has been associated with several morbidities, such as a higher incidence of infection, donor site pain, hematoma development, increased operative time, and blood loss (Younger and Chapman, 1989). Consequently, a variety of alternatives have been developed and investigated. Alternative grafts are usually classified as extender, enhancer, or substitute grafts (Tavares et al., 2022). An extender decreases the need for large amounts of autologous bone grafting while offering the same bone formation properties as autologous bone from the iliac crest. An enhancer is a material combined with autologous bone to increase successful fusion rates compared to autologous bone alone. A substitute replaces an autologous bone graft and presents the same or higher healing success rates. These materials are often assembled in various proportions to achieve spinal fusion. These options include allografts, synthetic grafts, and growth factors. Furthermore, autologous cell therapies with osteoinductive potential, such as BMA, mesenchymal stem cells (MSCs), as well as platelet products, have been incorporated into regenerative regimens to enhance spinal fusion rates (Feng et al., 2020; Salamanna et al., 2024).

Allogeneic bone has been widely used as an alternative to autogenous graft in orthopedic surgery. However, concerns have been raised regarding its reduced osteogenic potential and historically documented, albeit currently very low, risk of disease transmission (Baldwin et al., 2019). Thus, other biomaterials, such as demineralized bone matrix (DBM), bone marrow aspirate (BMA), bone morphogenetic protein (BMP), and various bioceramics have been studied for spinal fusion, and have demonstrated clinically favorable results when used as bone graft substitutes or extenders (Morris et al., 2018).

In general, bioceramics are the best-studied bone substitute materials (Sarkar and Lee, 2015). The term bioceramics comprises a range of biocompatible inorganic non-metallic materials, including calcium phosphates, such as Hydroxyapatite (HA) and Beta-tricalcium phosphate (ß-TCP), or newer developments such as silicate bioceramics (Brunello et al., 2020). Several of these scaffolds were demonstrated to be osteoconductive in animal models and showed better results than leaving the bone defects with no grafting material (Brunello et al., 2020). Most commonly, bioceramics are combined with autograft material, such as local autograft, autograft from the iliac crest, and/or BMA. The overall fusion rate for ceramic-based grafts results to be 86%, and the specific type of ceramic used did not significantly influence the fusion rate (Nickoli and Hsu, 2014; Plantz et al., 2021).

The optimal synthetic bone substitute should be osteoconductive, osteoinductive and resorbable. It should be possibly traceable *in vivo*; to this aim, radiolucency is ideal for optimal radiographic assessment. In addition, the ideal bone substitute should be thermally nonconductive, sterilizable, and readily available at a reasonable cost (Pryor et al., 2009).

All the above issues strongly support the effort to develop new materials with the ambition to overcome existing limitations and provide a real, new breakthrough solution to this challenge. b.Bone (manufactured by GreenBone Ortho SpA, Italy) is a ceramic reabsorbable scaffold with a geometrical structure, engineered to reflect anatomical and physiological bone hierarchical structures. b.Bone is produced through a biomorphic transformation process that preserves the three-dimensional hierarchical architecture of the original natural template (Tampieri et al., 2019). The rattan wood precursor exhibits a highly organized hierarchical structure characterized by interconnected channels and multiscale porosity, closely resembling the structural organization of natural bone. Through biomorphic conversion, this native architecture is retained and transformed into a calcium phosphate scaffold with a bone-like hierarchical framework extending from the macro-to the nanoscale (Sprio et al., 2021; Bigoni et al., 2020). The resulting scaffold maintains an interconnected porous network derived from the original rattan structure, closely resembling the organization of cancellous bone, and presents the following properties.

**Table:** 

Property	​
Total porosity	≥45 vol.%
Interconnected porosity	≥45%
Macropore size	(>10 µm) > 30%
Microporosity	(<10 µm) > 30%
Composition	HA = 85% ß-TCP = 15% Ca/P = 1,60 with presence of Mg2+ and Sr2+ ions
Compressive strength	20–26 MPa
Resorption	Gradual, coupled with bone remodeling

Unlike conventional synthetic calcium phosphate grafts, which are generally manufactured by powder compaction and sintering, the chemistry of b.Bone consists of nanostructured Mg/Sr-doped hydroxyapatite and β-tricalcium phosphate. Differently from high temperature sintering processes, the biomorphic transformation process uniquely allows to retain ion doping in the hydroxyapatite phase, thus obtaining very high bioactivity combined with the controlled resorption characteristics of β-TCP. This nanostructure provides a favorable interface for protein adsorption, cell attachment, and osteogenic cell proliferation, thereby supporting cellular colonization and early bone-forming activity. The preserved interconnected pore architecture facilitates vascular invasion, cell migration, nutrient diffusion, and bone ingrowth, while the intrinsic microporosity further increases the available surface area for biological interactions (Panseri et al., 2021). The presence of magnesium and strontium ions may further contribute to the biological performance of the scaffold by modulating osteogenic responses and bone remodeling processes (Kon et al., 2021). These structural and compositional features are intended to promote progressive scaffold remodeling through coupled resorption and new bone formation, thereby supporting physiological bone regeneration.

*b.Bone* is suitable for the surgical procedures for repair of long load-bearing bones (as segment defects in long bones) as well as for repair of defects in non-loaded bones. We report here the results of a feasibility clinical investigation aimed to evaluate b.Bone granules as bone graft extender in posterolateral fusion treatment of degenerative thoracolumbar, lumbar or lumbosacral spinal conditions, in terms of preliminary performance and safety.

## 2 Materials and methods

### 2.1 Study design

This is a prospective, open-label, single-arm clinical investigation conducted in two European clinical centers. A total of 16 patients qualified for posterolateral spinal fusion of one to eight levels in the thoracolumbar and lumbosacral region (T10-S1) have been recruited and enrolled. The study was approved by the Ethics Committee of the Coordinating center (prot. 1355, 26/01/2023), by the Ethics Committee of the Ärztekammer Saxonia Anhalt, by the Italian Ministry of Health and by the BfArM (Federal Institute for Drugs and Medical Devices, in Germany). The study was registered in Clinicaltrial.gov with number NCT05906394.

The study was conducted according to Helsinki declaration, and all patients signed a study-specific informed consent.

The primary objective of this study was to evaluate the performance of b.Bone through the degree of radiographic fusion at 12 months after implantation in patients requiring posterolateral fusion treatment due to degenerative spinal conditions at the thoracolumbar, lumbar, or lumbosacral levels.

Secondary objectives were to assess neurological function, patient-reported outcomes (back and lower limb pain, ODI, EQ-5D-5L), and evaluate the safety.

Patients were recruited according to the following inclusion and exclusion criteria.

#### 2.1.1 Inclusion criteria

1. Male or female patient ≥18 years old.
2. Patients requiring posterolateral fusion treatment for degenerative thoracolumbar, lumbar, or lumbosacral spinal conditions up to eight levels.
3. Patients willing and able to attend the follow-up visits and procedures foreseen by the study protocol.
4. Patients who have provided consent to the processing of personal data.

#### 2.1.2 Exclusion criteria

1. Any previous surgical attempt(s) for spinal fusion (revision surgery) of the intended segment(s).
2. Patients who have been treated with chemotherapy or radiotherapy within 12 months before the enrolment.
3. Indication for spinal fusion because of an acute traumatic reason, like a spinal fracture.
4. Spinal infection.
5. Spinal tumour.
6. Patients currently treated with systemic immunosuppressive agents.
7. Patients who are currently enrolled in another clinical study that would directly interfere with the current study, except when the patient is participating in a purely observational registry with no associated treatments.
8. Body mass index (BMI) larger than 36 (morbidly obese).
9. Known severe osteoporosis.
10. Patients requiring instrumented fusion in the cervical spine.
11. Woman who is pregnant or breast-feeding.

The primary endpoint of the study was to determine the thoracolumbar/lumbosacral fusion rate by CT scan assessments at 12 months according to Brantigan-Steffee score (Brantiga et al., 1993) (Table 1).

**TABLE 1:** Brantigan-Steffee fusion classification.

Grade	Fusion results	Description
1	Obvious radiographic pseudoarthrosis	Pseudoarthrosis, collapse of construct, loss of disc height, vertebral slip, broken screw, displacement of the cage, resorption of bone graft
2	Probable pseudoarthrosis	Significant resorption of bone graft, major lucency or gap visible in the fusion area >2 mm
3	Radiographic status uncertain	A small lucency or gap may be visible with at least half of the graft area showing no lucency between the graft bone and the vertebral bone
4	Probable radiographic fusion	Bone bridges the entire fusion area with at least the density originally achieved at surgery. There should be no lucency between the graft bone and the vertebral bone
5	Radiographic fusion	The bone in the fusion area is more dense and more mature than originally achieved at surgery; there is no interface between the donor bone and the vertebral bone: a sclerotic line between the graft bone and the vertebral bone indicates solid fusion. Other indicators of solid fusion is fusion at facet joints and anterior progression of the graft in the disc

Successful fusion was defined as “probable radiographic fusion – 4” or “Radiographic fusion – 5” for at least one side (unilateral).

- For patients with PLF surgery with 2-level b.Bone, fusion is considered successful if both levels are fused (unilateral or bilateral).
- For patients with PLF surgery with 3-level b.Bone, fusion is achieved if it occurs in two out of three levels (unilateral or bilateral).

Unilateral posterolateral fusion, defined as a continuous intertransverse bony bridge on at least one side, is considered clinically acceptable in instrumented constructs. This is supported by CT-based diagnostic criteria (Carreon et al., 2007), radiographic classification systems (Christensen et al., 2001), and randomized trials (Lehr et al., 2020; Stempels et al., 2024), the latter powering its design based on an expected unilateral fusion rate of 50% and 70% side concordance.

The secondary endpoints were.

1. Fusion rate by CT scan assessments at 6 months according to the Brantigan-Steffee score.
2. Back and Leg pain assessed by Visual Analog Scale (VAS) scores at 6 weeks, 3, 6, and 12 months post-treatment.
3. Functional activity assessed by the Oswestry Disability Index (ODI) Questionnaire at 6 weeks, 3, 6, and 12 months post-treatment.
4. Quality of life assessed by the EQ-5D-5L Questionnaire at 6 weeks, 3, 6, and 12 months post-treatment.
5. Neurological function based on evaluation of motor, sensory, reflex and straight leg raise tests at 3, 6, and 12 months post-treatment.
6. Safety: Adverse Events collected and evaluated through the clinical trial period. Classification of spine surgery AEs according to SAVES-V2 “Spine Adverse Events Severity System”, ver. 2.0 (Rampersaud et al., 2016).

### 2.2 Surgical procedure and data collection

Patients enrolled for this clinical trial underwent up to a three-level posterolateral fusion (PLF) procedure using b.Bone as a graft extender in combination with autologous bone in a 1:1 ratio based on volume. Equal amounts of autologous bone and b.Bone granules were measured using a syringe without needle. Autologous bone was harvested from decompression (complete or partial laminectomy) and/or from the decortication of all the available cortical surfaces, including the dorsal, superior and inferior surfaces of the transverse processes. The PLF surgical procedure was performed as per standard of care at the investigation site.

No control was used in this feasibility clinical investigation; b.Bone was mixed with autologous local bone (bone obtained from decompression and decortication), so that no extraction of autologous bone material from another anatomical site was performed, which might involve complications for the donor site.

Patients were evaluated at discharge, and after 6 weeks, 3 months, 6 months, and 12 months with X-ray, CT scans and physical assessment. The study was completed when the last patient completed the last follow-up visit.

The fusion assessment was performed on CT scans obtained at 6- and 12-month follow-up, analyzing multiplanar reconstructions. The CT scans were evaluated by the investigators, and bone fusion results were entered into the eCRF.

The raw volume dataset (DICOM scans) and all reconstructions were de-identified and transferred by the clinical sites to CoreLab Avania (Barcelona, Spain) for assessment by an independent radiologist, who prepared specific reports for each patient and time point.

For cases where there was disagreement on the overall patient fusion between the site investigators and the CoreLab’s assessments, an external independent auditor (additional radiologist) was appointed to re-read the selected imaging cases. The results of the independent audit were documented in specific reports.

### 2.3 Patient-reported outcomes

Patient-Reported Outcomes were collected at baseline and then at 6 weeks, 3 months, 6 months and 12 months follow-up.

Pain intensity was measured using a 10-point Visual Analog Scale, where 0 represents “no pain” and 10 represents “worst pain imaginable”. Three types of pain were registered: back pain, right leg pain, and left leg pain.

The Oswestry Disability Index (ODI) questionnaire was used to measure patient’s level of disability for low back pain. This patient-completed questionnaire provides a subjective percentage score of the patient’s functional status in various activities of daily living. The lower the percentage, the higher the patient’s functionality. 0%–20% minimal disability, 80%–100% significant disability or bedridden.

The EQ-5D-5L questionnaire was administered to patients to assess their health-related quality of life across five dimensions: mobility, self-care, usual activities, pain/discomfort, and anxiety/depression. Each dimension has five levels of severity: no problems, slight problems, moderate problems, severe problems, and extreme problems.

The EQ-5D-5L index value (overall health utility score) was calculated from the responses to the five dimensions, based on country-specific value sets, and reflects the overall health status of the individual. The index scores ranges from 0 (worst possible health state) to 1 (best possible health state).

Patients also rated their overall health on a scale from 0 (worst health imaginable) to 100 (best health imaginable), providing a snapshot of their perceived health status on the day of assessment.

#### 2.3.1 Clinical outcomes: neurological function

Neurological function was assessed based on evaluations of motor, sensory, reflex and straight leg raise tests at 3, 6, and 12 months post-treatment. Neurological success was defined as maintenance or improvement of neurological function postoperatively compared to baseline (before the procedure).

### 2.4 Statistical analysis

Continuous variables were summarized as mean ± standard deviation (SD) if normally distributed, or as median and interquartile range (IQR) if not normally distributed. Normality was assessed using the Shapiro–Wilk test. Categorical variables were described through absolute (n) and relative (%) frequencies.

The number of patients with available information and missing data was presented.

Change from baseline was calculated as follows:

Change from baseline:Value at visit−Value at baseline

Percent change from baseline was calculated as follows:

$$Percent\;change\;from\;baseline:\left(\frac{Value\;at\;visit-Value\;at\;baseline}{Value\;at\;baseline}\right)*100$$

A threshold of p < 0.050 was considered statistically significant.

Analyses were performed in SAS v9.4 under SAS Enterprise Guide v8.3.

## 3 Results

As reported in Figure 1, 16 patients were recruited in the two clinical centers and treated with b.Bone, according to the study protocol; however, one patient was withdrawn postoperatively from the study because of a revision surgery with graft removal occurring 24 days after the surgery.

**FIGURE 1:**
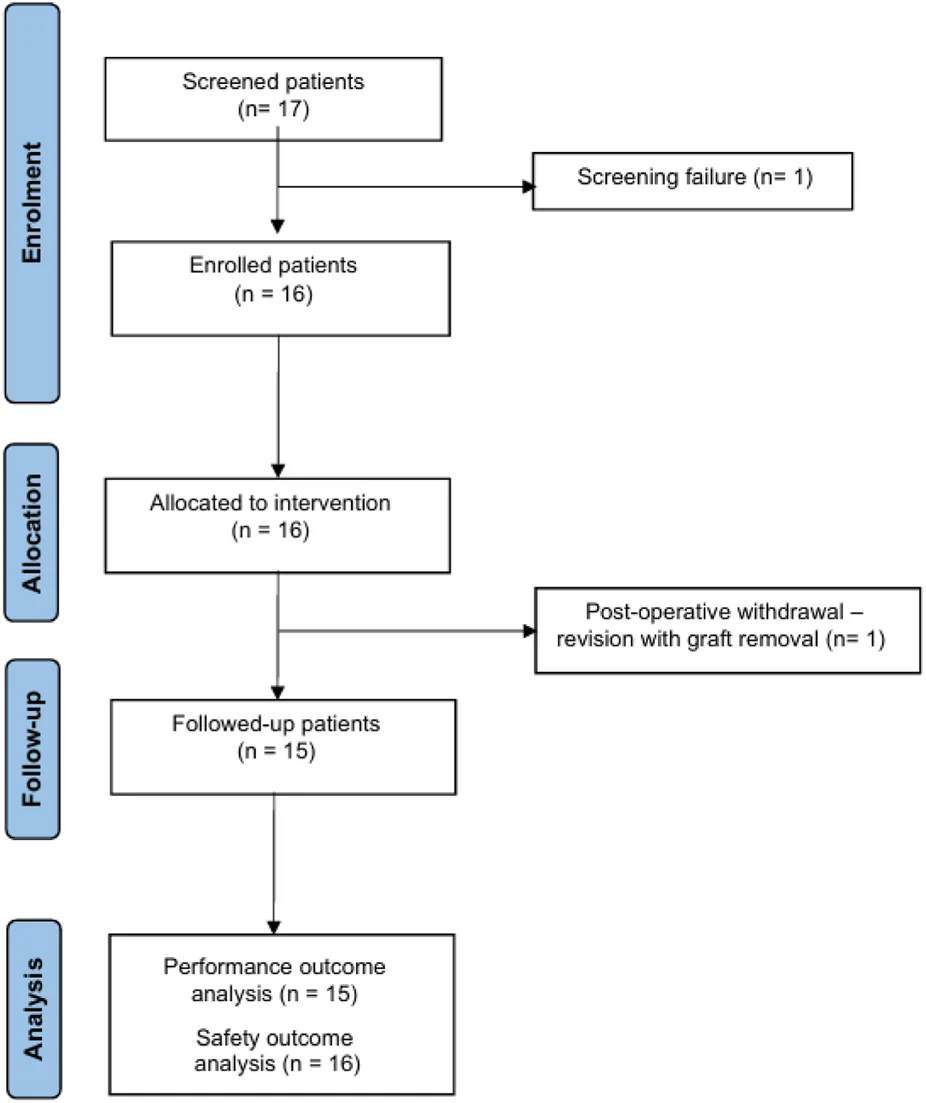
Study patients’ flow.

Patients between 19 and 74 years of age were enrolled in the study, with a mean age of 57.1 years. Of the 15 participants who completed the clinical trial, eight were men and seven were women. History of smoking was reported by one patient. Details of demographic characteristics are presented in Table 2.

**TABLE 2:** Demographic and smoking characteristics.

​	Population (N = 15)
Sex	
N	15
Female	7 (46.7)
Male	8 (53.3)
Missing	0
Age (years)	
N	15
Mean (SD)	57.07 (14.75)
Median (Q1; Q3)	58.00 (52.00; 70.00)
Min; Max	19.00; 74.00
Missing	0
Ethnicity	
N	15
White	14 (93.3)
Black	1 (6.7)
Asian	0 (0.0)
Other	0 (0.0)
Missing	0
Current smoker	
N	15
No	14 (93.3)
Yes	1 (6.7)
Missing	0

As reported in Table 3, common diagnoses among the patients included degenerative disc disease, adult deformity, stenosis, and spondylolisthesis. Ten patients presented multiple spinal conditions. All patients referred for surgery had long-lasting symptoms, in particular back pain and leg pain. Concerning the work status, most patients were not working or were restricted in working activities. Most patients were following pharmacological or conservative treatment, while four patients (26.7%) had already undergone surgical treatment.

**TABLE 3:** Spinal disease history and diagnosis for spinal fusion.

​	Population (N = 15)
Duration of back pain (months)	
N	14
Mean (SD)	113.93 (139.28)
Median (Q1; Q3)	90.00 (21.00; 144.00)
Min; Max	12.00; 540.00
Missing	1
Duration of left leg pain (months)	
N	11
Mean (SD)	26.45 (34.02)
Median (Q1; Q3)	12.00 (9.00; 36.00)
Min; Max	0.00; 120.00
Missing	4
Duration of right leg pain (months)	
N	8
Mean (SD)	13.88 (14.41)
Median (Q1; Q3)	11.50 (8.00; 12.00)
Min; Max	0.00; 48.00
Missing	7
Current work status	
N	15
Not working	6 (40.0)
Working full-time (32+ h/week)	6 (40.0)
Working part-time (8–31 h/week)	1 (6.7)
Not applicable (retired, student, disabled)	2 (13.3)
For patients currently working, restriction in work activity	
N	7
Unrestricted work activity	4 (57.1)
Restricted work activity	3 (42.9)
Missing	0
Current activity level	
N	15
Light	2 (13.3)
Moderate	11 (73.3)
Vigorous	2 (13.3)
Brace use	
N	15
No	12 (80.0)
Yes	3 (20.0)
Previous treatments for the same pathology	
N	15
None	1 (6.7)
Surgical	4 (26.7)
Pharmacological	3 (20.0)
Conservative	7 (46.7)
Diagnosis indication for spinal fusion	
N (%)	15 (100.0)
Deformity	10 (66.7)
Spondylolisthesis	5 (33.3)
Stenosis	8 (53.3)
Degenerative disc disease	11 (73.3)

Table 4 provides a comprehensive overview of posterolateral fusion surgery with b.Bone device implantation performed on 15 patients. Most patients had multiple spinal levels involved, with two levels (33.3%) and eight levels (33.3%) being the most common. 40% of patients had thoracolumbar spinal tracts treated, with specific levels such as T4–T5, T5–T6, T8–T9, and T9–T10 being the most frequently treated. 60% of patients had lumbo-sacral spinal tracts treated, with L4–L5 (46.7%) and L3–L4 (40.0%) being the most common levels treated. Only one patient (6.7%) underwent hemi-laminectomy, while the majority (93.3%) did not require decompression at levels treated with b.Bone. All patients (100%) had rods and screws implanted, with additional instrumentation such as intervertebral cages used in 53.3% of cases.

**TABLE 4:** Posterolateral fusion surgery with b.Bone implantation.

​	Population (N = 15)
Number of total spinal levels involved	
N	15
Single level	0 (0.0)
2 levels	5 (33.3)
3 levels	3 (20.0)
4 levels	0 (0.0)
5 levels	0 (0.0)
6 levels	1 (6.7)
7 levels	0 (0.0)
8 levels	5 (33.3)
9 levels	1 (6.7)
Thoracolumbar spinal tracts treated with b.Bone	
N (%)	6 (40.0)
T1–T2	0 (0.0)
T2–T3	0 (0.0)
T3–T4	0 (0.0)
T4–T5	2 (13.3)
T5–T6	2 (13.3)
T6–T7	1 (6.7)
T7–T8	1 (6.7)
T8–T9	2 (13.3)
T9–T10	2 (13.3)
T10–T11	2 (13.3)
T11–T12	1 (6.7)
T12–L1	0 (0.0)
Lumbo-sacral spinal tracts treated with b.Bone	
N (%)	9 (60.0)
L1–L2	0 (0.0)
L2–L3	3 (20.0)
L3–L4	6 (40.0)
L4–L5	7 (46.7)
L5–S1	5 (33.3)
Decompression at levels treated with b.Bone	
N	15
None	14 (93.3)
Hemi-laminectomy	1 (6.7)
Decompression side	
N	1
Right	1 (100.0)
Left	0 (0.0)
Left and right	0 (0.0)
Instrumentation used	
N (%)	15 (100.0)
Rod	15 (100.0)
Screws	15 (100.0)
Other	8 (53.3)
Other instrumentation used detail	
N	8
1 cage L3/4, PEEK (polyetheretherketone)	1 (12.5)
1 cage L5/S1, titanium	2 (25.0)
1 cage, L3/4, titanium	1 (12.5)
2 cages L2/3, L4/5, titanium	1 (12.5)
2 cages L4/5, L5/S1, titanium	1 (12.5)
2 cages, L3/4, L4/5, polyetheretherketone	1 (12.5)
2 cages, L4/5, L5/S1, PEEK (polyetheretherketone)	1 (12.5)
Estimated blood loss (mL/cc)	
N	15
Mean (SD)	740.00 (343.93)
Median (Q1; Q3)	700.00 (500.00; 800.00)
Min; Max	250.00; 1,500.00
Perioperative blood transfusion	
N	15
No	8 (53.3)
Yes	7 (46.7)
Bone quality	
N	15
Good	13 (86.7)
Poor	0 (0.0)
Osteopenia	2 (13.3)
Osteoporotic	0 (0.0)
Sclerotic	0 (0.0)
Intraoperative complications	
N	15
No	12 (80.0)
Yes	3 (20.0)
Type of complications	
N	3
Dural tear	3 (100)

The mean estimated blood loss was 740 mL, with a range from 250 mL to 1,500 mL. 46.7% of patients required blood transfusions during the perioperative period. The majority of patients had good bone quality (86.7%), with some cases of osteopenia (13.3%).

Three patients (20%) experienced intraoperative complications, specifically dural tears.

### 3.1 Spinal fusion assessment

In Table 5, the results of spinal fusion at 6 months follow-up for levels treated with b.Bone are summarized. Successful radiographic fusion was achieved in 10 out of 15 patients, representing a 66.7% success rate.

**TABLE 5:** Fusion assessment at 6 months follow up.

Case number (N = 15)	Spinal levels treated with b.Bone	Overall fusion 6 months
01	L2–L3	Successful fusion
	L3–L4	
02	T10–T11	Not yet achieved
03	T4–T5	Successful fusion
	T5–T6	
05	T4–T5	Successful fusion
	T5–T6	
	T6–T7	
06	T7–T8	Successful fusion
	T8–T9	
07	T8–T9	Not yet achieved
	T9–T10	
08	T9–T10	Successful fusion
	T10–T11	
	T11–T12	
09	L4–L5	Successful fusion
	L5–S1	
10	L2–L3	Not yet achieved
	L3–L4	
11	L3–L4	Successful fusion
	L4–L5	
	L5–S1	
12	L3–L4	Successful fusion
	L4–L5	
	L5–S1	
13	L3–L4	Not yet achieved
	L4–L5	
14	L4–L5	Successful fusion
	L5–S1	
15	L4–L5	Not yet achieved
	L5–S1	
16	L2–L3	Successful fusion
	L3–L4	
	L4–L5	

According to the study protocol, patients 02, 07, 10, 13, 15 had no successful fusion at 6 months FU.

The radiographic spinal fusion results at 12 months follow-up for levels treated with b.Bone are summarized in Table 6, detailing the overall fusion assessment for each participant. According to the study protocol, all cases (N = 15) achieved successful radiographic fusion at all spinal levels treated with b.Bone within 12 months post-surgery. This represents a 100% rate of successful radiographic spinal fusion.

**TABLE 6:** Fusion assessment at 12 months follow up.

Case number (N = 15)	Spinal levels treated with b.Bone	Overall fusion 12 months
01	L2–L3	Successful fusion
	L3–L4	
02	T10–T11	Successful fusion
03	T4–T5	Successful fusion
	T5–T6	
05	T4–T5	Successful fusion
	T5–T6	
	T6–T7	
06	T7–T8	Successful fusion
	T8–T9	
07	T8–T9	Successful fusion
	T9–T10	
08	T9–T10	Successful fusion
	T10–T11	
	T11–T12	
09	L4–L5	Successful fusion
	L5–S1	
10	L2–L3	Successful fusion
	L3–L4	
11	L3–L4	Successful fusion
	L4–L5	
	L5–S1	
12	L3–L4	Successful fusion
	L4–L5	
	L5–S1	
13	L3–L4	Successful fusion
	L4–L5	
14	L4–L5	Successful fusion
	L5–S1	
15	L4–L5	Successful fusion
	L5–S1	
16	L2–L3	Successful fusion
	L3–L4	
	L4–L5	

If only bilateral fusion was considered, a total of 31/34 levels (91.2%) resulted to be fused 12 months after surgery and 12 out of 15 patients (80%) achieved bilateral fusion at 12 months follow up (Table 7).

**TABLE 7:** Assessment of bilateral fusion.

Metric	Value %	Counts	Findings
Overall level fusion rate	91.18	Total levels = 34 Fused levels = 31	91.2% of treated spinal levels achieved bilateral fusion (grades 4 or 5 on both sides) at 12 months
Overall patient fusion rate	80.00	Total patients = 15 Fused patients = 12	80% of patients had all treated levels fused bilaterally at 12 months

For patients having additional instrumented spinal levels fused beside those treated with b.Bone, the fusion assessment was also performed for these additional levels, and results are reported in the Supplementary Material file (Table 1). Across the evaluated cases, levels treated with b.Bone generally showed higher fusion grades at 6 and 12 months compared with the adjacent levels treated without b.Bone, although the number of assessable non–b.Bone levels was limited due to anatomical or surgical constraints.

One representative case is reported in Figure 2. X-ray imaging shows the pre-operative spinal condition of the patient and the surgical correction with rods and screws instrumentation from T9–L5. CT scans performed 12 months after surgery show intertransverse fusion in multiplanar reconstructions at T9–T12 levels treated with b.Bone. The fusion was achieved bilaterally at all treated levels.

**FIGURE 2:**
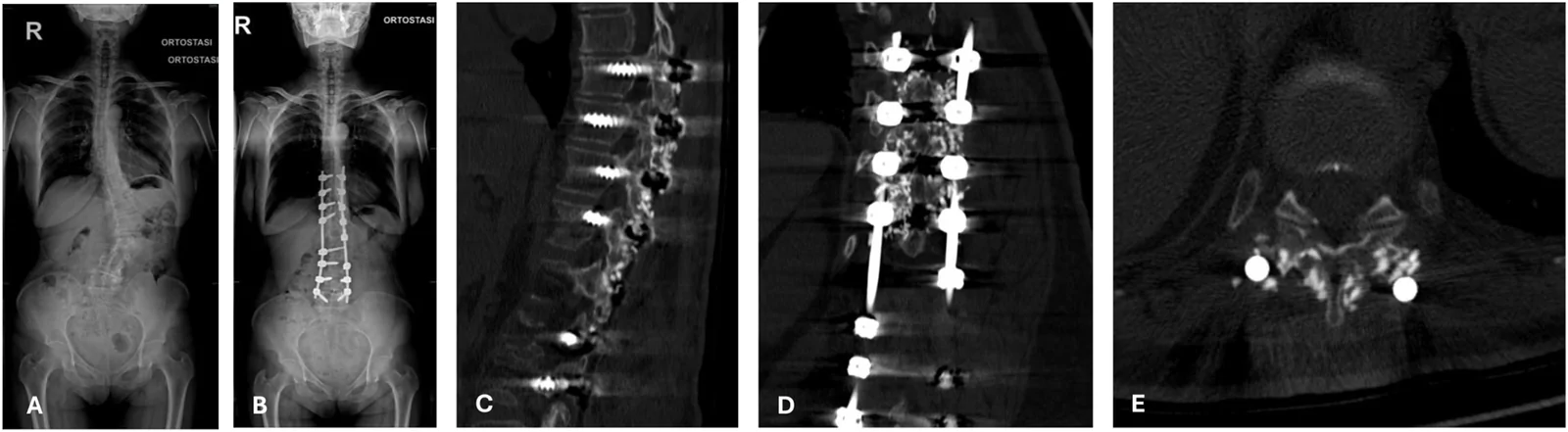
Radiographic imaging of a representative case. **(A)** pre-operative X-ray; **(B)** post-operative X-ray; **(C–E)** CT scans at 12 months follow up (sagittal, coronal and axial reconstructions).

### 3.2 Patient reported outcomes

All 15 patients experienced back pain before surgery, six patients reported also right leg pain and 11 patients reported also left leg pain. In Figure 3, VAS scores for back, right and left legs at baseline and during the follow up period are reported.

**FIGURE 3:**
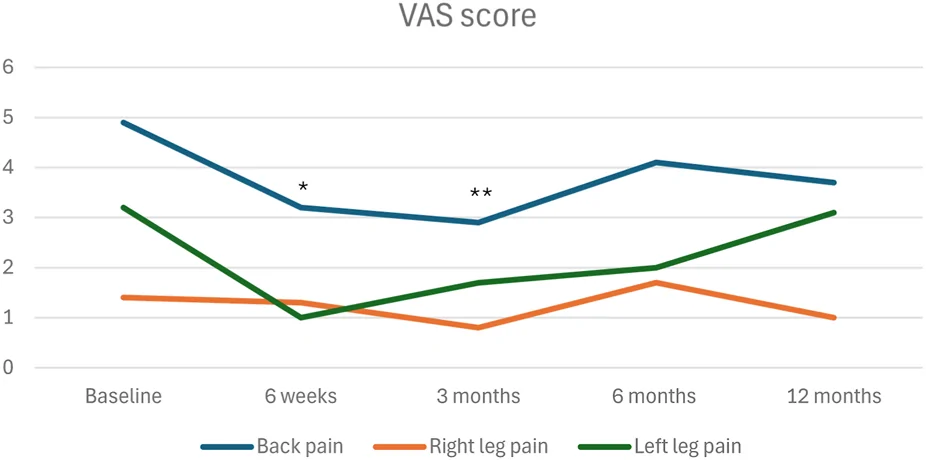
12-Month Trends on Back, Right Leg, and Left Leg Pain Scores. *The difference between baseline score and FU Visit 1 is statistically significant (p = 0.034). **The difference between baseline score and FU Visit 2 is statistically significant (p = 0.007).

The mean back pain score at baseline (4.9 ± 2.8) was higher with respect to right and left leg pain scores. We observed a significant improvement in back pain after surgery, starting from 6 weeks follow-up and increasing at 3 months follow-up, then the mean back pain score was maintained below the baseline value until the final follow-up.

The improvement of right and left leg pain after surgery was not statistically significant, but it should be considered that the baseline values were low.

Figure 4 illustrates the mean Oswestry Disability Index (ODI) percentage scores over a 12-month period. No significant differences were observed between baseline and follow-up visits and the patients’ functional ability remained stable over time.

**FIGURE 4:**
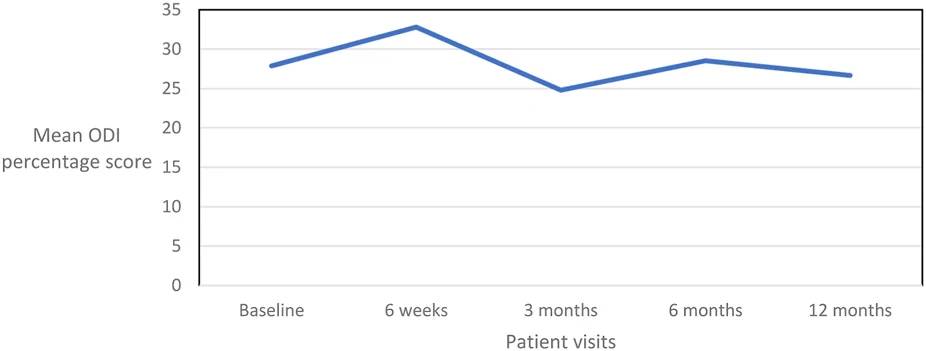
12-Month trends in disability levels based on ODI scores.

Figure 5 reports the mean EQ-5D-5L index scores over time. No significant differences were observed between baseline and follow-up visits, even if there was a trend of improvement after surgery, which remained stable over time.

**FIGURE 5:**
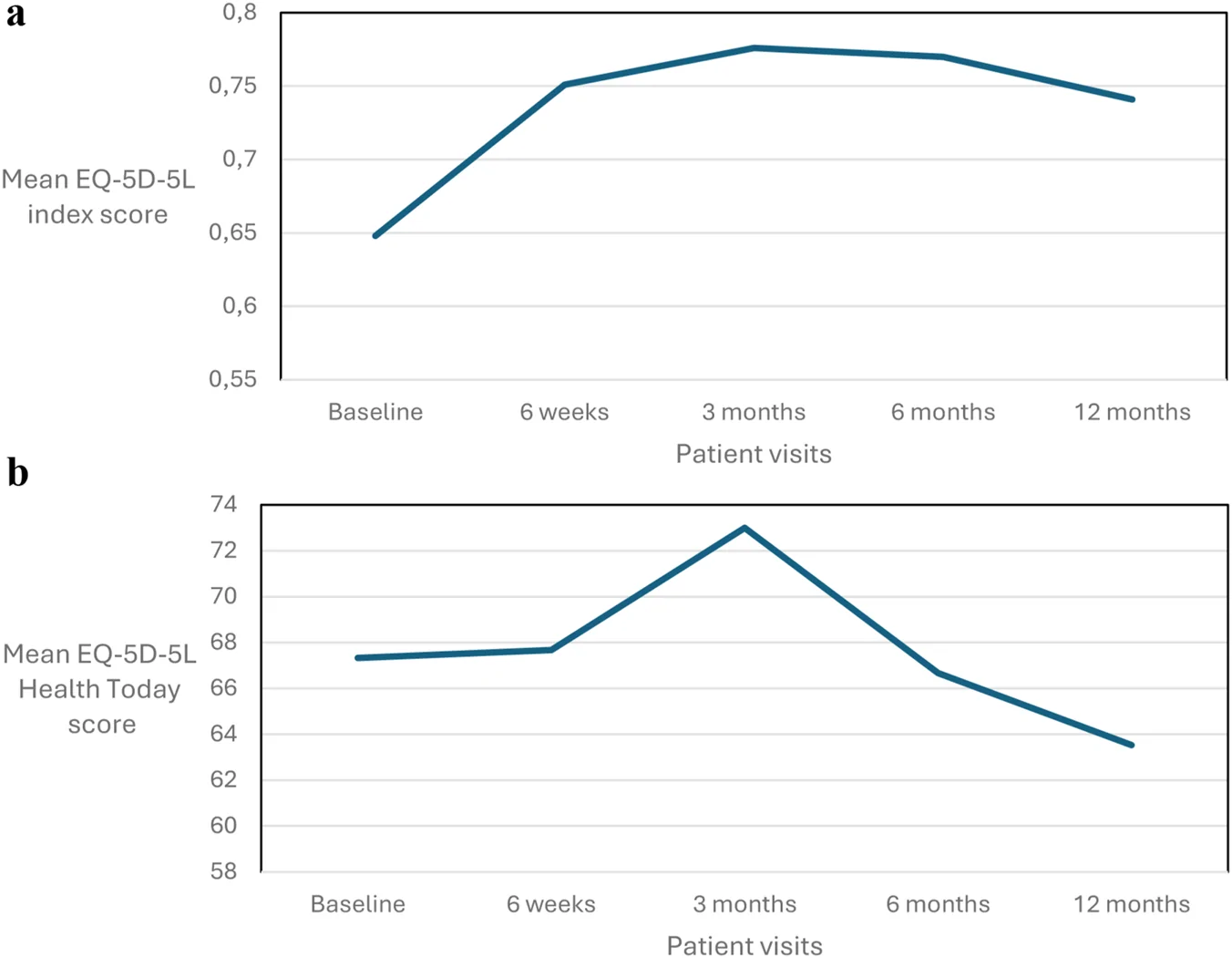
**(a)** 12-Month trends in health-related quality of life based on EQ-5D-5L. **(b)** Trends in perceived health status over 12 months based on EQ-5D-5L.

Figure 5 represents the trend of EQ-5D-5L Health Today scores at baseline and follow-up visits over a 12-month period. No significant differences were observed between baseline and follow-up visits. An improvement was recorded at 3 months follow-up, with a subsequent decrease over time. It should be noted that the patients’ population started from a high perceived health status.

### 3.3 Clinical outcomes: neurological function

At baseline, 86.7% of patients had normal motor function, while 13.3% had abnormal motor function. In terms of motor function evolution from baseline, 6.7% of patients showed improvement at both the 3-month and 6-month follow-ups. Most patients (86.7% at 3 months and 80.0% at 6 months) maintained their motor function.

Tables 8, 9 summarize the motor function outcomes at baseline, 3 months, 6 months, and 12 months post-treatment. At 12-month follow-up, all patients (100.0%) maintained their motor function, with no cases of deterioration.

**TABLE 8:** Motor function status at each follow-up visit.

​	Baseline N = 15	FU Visit 2 (3 months) N = 15	FU Visit 3 (6 months) N = 15	FU Visit 4 (12 months) N = 15
Motor function				
Normal	13 (86.7%)	13 (86.7%)	12 (80.0%)	13 (86.7%)
Abnormal	2 (13.3%)	2 (13.3%)	3 (20.0%)	2 (13.3%)
Missing value	0	0	0	0

**TABLE 9:** Motor function evolution from baseline to 3, 6 and 12 months.

​	Population (N = 15)
Motor function evolution from baseline to 3 months	
N	15
Improvement	1 (6.7)
Maintenance	13 (86.7)
Deterioration	1 (6.7)
Missing	0
Motor function evolution from baseline to 6 months	
N	15
Improvement	1 (6.7)
Maintenance	12 (80.0)
Deterioration	2 (13.3)
Missing	0
Motor function evolution from baseline to 12 months	
N	15
Improvement	0 (0.0)
Maintenance	15 (100.0)
Deterioration	0 (0.0)
Missing	0

At baseline, 86.7% of patients had normal sensory function, while 13.3% had abnormal sensory function. Tables 10, 11 summarize the sensory function outcomes at baseline, 3 months, 6 months, and 12 months post-treatment. At 1 year final follow-up, the baseline sensory function was maintained in 11 cases, improved in 2 cases and deteriorated in 2 cases.

**TABLE 10:** Sensory function status at each follow-up visit.

​	Baseline N = 15	FU visit 2 (3 months) N = 15	FU visit 3 (6 months) N = 15	FU visit 4 (12 months) N = 15
Sensory function				
Normal	13 (86.7%)	11 (73.3%)	14 (93.3%)	13 (86.7%)
Abnormal	2 (13.3%)	4 (26.7%)	1 (6.7%)	2 (13.3%)
Missing value	0	0	0	—

**TABLE 11:** Sensory function evolution from baseline to 3, 6 and 12 months.

​	Population (N = 15)
Sensory function evolution from baseline to 3 months	
N	15
Improvement	2 (13.3)
Maintenance	9 (60.0)
Deterioration	4 (26.7)
Missing	0
Sensory function evolution from baseline to 6 months	
N	15
Improvement	2 (13.3)
Maintenance	12 (80.0)
Deterioration	1 (6.7)
Missing	0
Sensory function evolution from baseline to 12 months	
N	15
Improvement	2 (13.3)
Maintenance	11 (73.3)
Deterioration	2 (13.3)
Missing	0

Concerning reflexes, 73.3% of patients had normal reflexes at baseline, while 26.7% of patients had abnormal reflexes. Tables 12, 13 summarize the reflexes outcomes at baseline, 3, 6, and 12 months post-treatment. At 1 year final follow-up, the baseline reflexes were maintained in 8 cases, improved in 4 cases and deteriorated in 3 cases.

**TABLE 12:** Reflexes status at each follow-up visit.

​	Baseline N = 15	FU visit 2 (3 months) N = 15	FU visit 3 (6 months) N = 15	FU visit 4 (12 months) N = 15
Reflexes				
Normal	11 (73.3%)	13 (86.7%)	11 (73.3%)	12 (80.0%)
Abnormal	4 (26.7%)	2 (13.3%)	4 (26.7%)	3 (20.0%)
Missing value	0	0	0	0

**TABLE 13:** Reflexes evolution from baseline to 3, 6 and 12 months.

​	Population (N = 15)
Reflexes evolution from baseline to 3 months	
N	15
Improvement	4 (26.7)
Maintenance	9 (60.0)
Deterioration	2 (13.3)
Missing	0
Reflexes evolution from baseline to 6 months	
N	15
Improvement	4 (26.7)
Maintenance	7 (46.7)
Deterioration	4 (26.7)
Missing	0
Reflexes evolution from baseline to 12 months	
N	15
Improvement	4 (26.7)
Maintenance	8 (53.3)
Deterioration	3 (20.0)
Missing	0

The straight leg raising test (Lasegue) was negative for 80% of patients at baseline for both the right leg and the left leg.

Table 14 reports the straight leg raising test outcomes for the left and right legs at 3, 6, and 12 months post-treatment with respect to baseline.

**TABLE 14:** Straight leg raising test from baseline to 3, 6 and 12 months - left and right legs.

​	Population (N = 15)
Left leg straight rising evolution from baseline to 3 months	
N	15
Improvement	3 (20.0)
Maintenance	12 (80.0)
Deterioration	0 (0.0)
Missing	0
Left leg straight rising evolution from baseline to 6 months	
N	15
Improvement	2 (13.3)
Maintenance	12 (80.0)
Deterioration	1 (6.7)
Missing	0
Left leg straight rising evolution from baseline to 12 months	
N	15
Improvement	3 (20.0)
Maintenance	11 (73.3)
Deterioration	1 (6.7)
Missing	0
Right leg straight rising evolution from baseline to 3 months	
N	15
Improvement	2 (13.3)
Maintenance	13 (86.7)
Deterioration	0 (0.0)
Missing	0
Right leg straight rising evolution from baseline to 6 months	
N	15
Improvement	2 (13.3)
Maintenance	13 (86.7)
Deterioration	0 (0.0)
Missing	0
Right leg straight rising evolution from baseline to 12 months	
N	15
Improvement	3 (20.0)
Maintenance	12 (80.0)
Deterioration	0 (0.0)
Missing	0

At 1 year final follow-up, the straight leg rising test at left was maintained in 11 cases, improved in 3 cases and deteriorated in 1 case, while the straight leg rising test at right was maintained in 12 cases and improved in 3 cases and no deterioration was detected.

At 1 year final follow-up, Lasegue test was positive only in one case for the left leg.

### 3.4 Adverse events

One of the secondary endpoints of this study was to assess the safety profile of b.Bone in patients undergoing posterolateral fusion treatment for degenerative thoracolumbar, lumbar, or lumbosacral spinal conditions.

As reported in Table 15, 27 adverse events (AEs) were reported during the clinical trial. Of these, 23 AEs occurred in the postoperative phase, and 4 were intraoperative. A total of 11 patients experienced at least one adverse event. Of the 27 adverse events documented, 11 (41%) occurred in two participants: one participant with a background of left hip arthrosis (a pre-existing condition) and previous decompression at the levels treated during this clinical trial; the second participant with a previous lumbar decompression and a background of persistent pain.

**TABLE 15:** Adverse events.

​	Population (N = 16)
Patients with at least one adverse event (AE)	11 (68.8%)
Total number of adverse events N	27
Intraoperative AEs	
Dural tear	3 (11.1%)
Vascular injury	1 (3.7%)
Post-operative AEs	
Construct failure with loss of correction	1 (3.7%)
Construct failure without loss of correction	2 (7.4%)
Deep vein thrombosis	1 (3.7%)
Deep wound infection	1 (3.7%)
Neurologic deterioration	2 (7.4%)
Other: cardiovascular disorder	1 (3.7%)
Other: disorder of blood parameters	1 (3.7%)
Other: gastrointestinal disorders	1 (3.7%)
Other: musculoskeletal disorder	3 (11.1%)
Other: numbness leg	1 (3.7%)
Other: persistent pain	2 (7.4%)
Other: postoperative anemia	1 (3.7%)
Other: recessional stenosis	1 (3.7%)
Other: side effects of pain medication	1 (3.7%)
Other: viral infection of the upper respiratory tract	2 (7.4%)
Other: wound edge necrosis	1 (3.7%)
Other: wound secretion	1 (3.7%)

The intraoperative AEs were: 3 cases of dural tears and 1 case of vascular injury. These AEs were resolved during the hospital stay without sequelae, the vascular injury requiring a longer hospitalization.

Major post-operative adverse events were: 2 cases of hardware failure, both occurring at 6 months follow-up and requiring revision surgeries; 1 case of deep wound infection requiring re-operation 24 days after surgery with debridement and removal of b.Bone implant (this patient was dropped out). Another case of construct failure without loss of correction (light screw mobilization) was registered, however re-intervention was not necessary. Minor AEs were registered after the surgery and were resolved without sequelae.

Hardware failure events and infection involved levels that were not treated with b.Bone, so no direct association of AEs with the device was demonstrated. No device deficiencies were reported.

### 3.5 Further follow-up data

After the study period, further follow-up data were collected in one center for seven patients. One patient required revision surgery 20 months after the index procedure (T4–L2 stabilization) due to distal junctional failure at (L2–L3), necessitating extension of instrumentation. This event was not device-related, as the b.Spine graft was implanted at T4–T7 tract. At the time of the revision surgery, a CT scan confirmed successful fusion at the b.Spine–treated levels. Another patient died 2 years after the index surgery (stabilization T10-S1-ileum) due to the diffusion of a spondylodiscitis at cervical level. This event was also not associated with the device. Radiological assessments performed approximately 24 months after the index surgery showed no signs of complications and confirmed successful fusion in the remaining five patients. Patients treated at the second center were not followed beyond the study period (12 months). However, since their last study visits, no patient-reported complications have been received to date.

## 4 Discussion

Autologous bone graft from locations such as the iliac crest is still considered the gold standard for bone regeneration in spinal surgery as it comprises extracellular matrix (osteoconduction), growth factors (osteoinduction) and cells (osteogenesis). The drawbacks of this treatment are sustained complications at the donor site (in more than 20%), including pain, hematoma, infection, and nerve injury. Harvesting the bone from the iliac crest also increases the duration of surgery and might prolong the hospital stay (Tavares et al., 2022; Younger and Chapman, 1989; Feng et al., 2020; Salamanna et al., 2024).

Synthetic bone substitutes and extenders provide an osteoconductive environment, prevent donor site morbidity, local infection, and ideally induce no reactive inflammatory or immunogenic response from local recipient tissue. They incorporate a multitude of graft materials including ceramic extenders, HA, ß-TCP, Ca sulphate, biphasic Ca phosphate, polylactic/glycolic acid, and carboxymethylcellulose (Garimella et al., 2024; De Pace et al., 2025). According to a meta-analysis of Morris et al. (Morris et al., 2018) about bone grafts for posterolateral fusion, 45 studies looked at iliac crest bone grafts, in conjunction with local autograft (29), biphasic calcium phosphate (1), autologous platelet concentrate (2), bone morphogenetic protein (BMP) (6), or demineralized bone matrix (DBM) (1). 18 studies assessed fusion rate by plain radiographs alone (37.5%), while six used CT scan (12.5%), and 24 used both (50.0%). Aggregate mean fusion rates among these ranged from 68.0% to 91.5%. Another 22 studies evaluated fusion rates of local autograft, either isolated (3) or combined with ceramic extenders (8), DBM (4), BMP (1), BMA (4), autologous platelet concentrate (1), or iliac crest bone graft (1). Aggregate mean fusion rate ranged from 75% to 95.5%. With the exception of studies involving allograft (mean fusion rate 40.0%), the mean fusion rate for all other graft combinations exceeded 70.0%. The meta-analysis only included studies with a minimum follow-up of 12 months (Morris et al., 2018).

Focusing the literature research on selected benchmark synthetic ceramic bone substitutes used for posterolateral fusion, we found nine clinical studies, which are summarized in the Supplementary Material file (Table 2) (Lehr et al., 2020; Stempels et al., 2024; Kanter et al., 2016; Epstein, 2006; Epstein, 2009; Lerner et al., 2009; Zanotti et al., 2021; Giorgi et al., 2015; G et al., 2022). These publications provide strong evidence supporting the clinical performance and safety of synthetic bone graft substitutes in posterolateral spinal fusion, both as standalone grafts and as extenders to autograft. Across studies, fusion rates at 12 months follow up range from approximately 55.0%–95.0%, with a mean fusion rate of about 81.2% (N = 469). Several devices demonstrate non-inferiority or superiority to autograft. Long-term follow-up (up to 4 years) confirms sustained fusion and clinical improvement. Current evidence suggests their optimal use is as graft extenders rather than stand-alone substitutes.

Overall, the risk profile was comparable to autograft, with no signal of increased complications rate. In fact, ceramic extenders reduce donor site morbidity associated with iliac crest bone graft harvesting. Most reported complications were typical of instrumented spinal fusion (infection, hardware issues, nerve irritation), not specifically caused by the graft material.

b.Bone is a new generation ceramic resorbable scaffold with a geometrical structure, engineered to reflect anatomical and physiological bone hierarchical structures and made of biomimetic substituted calcium phosphate phases (HA and β-TCP and minerals).

Since b.Bone is a CE-marked device for use as a bone substitute for voids or gaps in the extremities and pelvis, the present feasibility clinical investigation was performed to capture performance and safety information of b.Bone granules when used outside their intended purpose in order to expand their indication to spinal fusion. b.Bone granules were used as a bone graft expander in posterolateral spinal fusion surgery in combination with autologous bone graft.

The clinical trial enrolled 16 patients in two different clinical centers, all patients underwent posterolateral fusion surgery for degenerative thoracolumbar, lumbar or lumbosacral spinal diseases. One patient dropped out the study 24 days after surgery. Clinical and radiographic data were collected at baseline before surgery and during follow-up periods, from 6 weeks to 12 months after surgery.

Taking into account the difficulty of distinguishing bone regeneration due to the biomaterial or autologous bone, we observed that, according to the study protocol, all 15 patients achieved radiographic spinal fusion at 1-year final follow-up with a 100% success rate. Considering bilateral fusion, 91.2% of spinal levels treated with b.Bone achieved bilateral fusion (grades 4 or 5 on both sides) at 12 months, and 12 patients (80%) had all treated levels fused bilaterally.

These overall spinal fusion rates position b.Spine within the performance range reported for synthetic bone graft substitutes in instrumented spinal fusion with a high rate of successful fusion 1 year after surgery. While these preliminary observations are encouraging, they should be interpreted with caution and further examined in routine clinical practice and post-market settings to better understand fusion outcomes across different treatment modalities and in a larger population.

From the clinical perspective, no case of neurological impairment was registered after surgery. The maintenance of baseline motor function was reported at 1-year final follow-up for all cases analysed, while sensory function was slightly deteriorated only in two patients, improved in two patients and stable in the other patients.

Patient-reported outcomes highlighted a significant improvement in back pain, which was evident from 6 weeks post-surgery, increased at 3 months, and was maintained over time. Other scores, including VAS for leg pain, ODI, and EQ-5D-5L, remained stable after the surgery, with no high levels at baseline.

These results suggest that the presence of b.Bone was not associated to a deterioration or worsening of neurological functions or patient-reported outcomes, while it could provide a potential contribution to the improvement of back pain after surgery.

Concerning safety assessment, the clinical trial demonstrated a safety profile of b.Bone which is consistent with existing treatments for spinal fusion. Out of 27 adverse events reported, 3 were major AEs requiring re-intervention. All the adverse events reported were typical of spinal fusion surgery; in particular, the incidence of hardware failure events causing revision surgery was not higher than that reported in literature (Puvanesarajah et al., 2016), and these AEs involved levels that were not treated with b.Bone, so no direct association of AEs with the device was demonstrated.

The favourable fusion outcomes observed in this study may also be interpreted in light of the unique structural and compositional characteristics of the biomaterial. Successful spinal fusion requires early vascularization, migration and attachment of osteogenic cells, and progressive replacement of the graft by newly formed bone. Through biomorphic transformation, b.Bone preserves the biomimetic chemistry of the natural bone as well as the hierarchical architecture of the rattan template, resulting in an interconnected multiscale porous network that supports tissue infiltration, vascular growth, and bone ingrowth. The biomimetic surface of b.Bone, characterized by high hydrophilicity, may enhance protein adsorption, cellular attachment, and osteogenic activity. This balanced structural and compositional profile differs from the conventional stoichiometric HA/TCP sintered ceramics, which often exhibit limited bioactivity and remodeling extent.

This pilot study has some limitations, in particular.

1. Sample Size: The study involved a relatively small sample size (N = 15), which may limit the generalizability of the results to a broader population.
2. Follow-Up Duration: The follow-up period was limited to 12 months, which may not capture long-term outcomes and potential late complications.
3. Patient Selection: The study participants had specific spinal conditions and pre-existing conditions, which may not represent the full spectrum of patients requiring spinal fusion surgery.
4. Lack of Control Group: The absence of a control group with other graft materials does not permit to demonstrate b.Bone statistical superiority or equivalence, even if non-inferiority was supported by comparison with literature data.
5. Combination of b.Bone with autologous bone: As the autologous bone possesses osteogenic, osteoinductive, and osteoconductive properties, the present study cannot distinguish whether the observed fusion outcomes were attributable to b.Bone or to the autograft itself.
6. Qualitative evaluation of spinal fusion (Brantigan scale): The evaluation of radiographic spinal fusion is a challenging issue, with different methods used for qualitative assessment, and the adoption of standardized quantitative methos would improve the quality of studies aiming to assess spinal fusion in the presence of biomaterials (Bilancia et al., 2025).
7. Presence of major adverse events requiring revision surgery: this can represent a limitation for the assessment of the safety of b.Bone, even if these AEs cannot be directly associated to the device.

Despite these limitations, the results of this clinical investigation contribute to the current scientific knowledge by demonstrating the safety and the non-inferiority of b.Bone as bone graft extender in multilevel posterolateral spinal fusion surgery, providing a reliable option for patients with degenerative spinal conditions, particularly in cases where autologous bone is poor or of low quality.

The positive outcomes of this clinical trial suggest several avenues for future research.

- Broader population studies: to investigate the safety and effectiveness of b.Bone in a larger and more diverse patient population, including spinal fusion approaches other than the posterolateral.
- Long-term follow-up: to conduct studies with extended follow-up periods to assess the long-term results.
- Comparative post-market studies: to compare the performance of b.Bone with other graft materials and techniques to establish its relative efficacy and safety.

These future studies will help to generalize the findings and confirm the broad applicability of b.Bone in spinal fusion surgeries, potentially leading to improved treatment protocols and patient outcomes.

## Data Availability

The raw data supporting the conclusions of this article will be made available by the authors, without undue reservation.
