# Supplementary material for: A pre-market, open-label, single-arm study to evaluate b.Bone for posterolateral fusion treatment of degenerative thoracolumbar, lumbar or lumbosacral spinal disease (b.Spine clinical trial)
Source: Front Bioeng Biotechnol. 2026 Sep 25;14:1851820. doi: 10.3389/fbioe.2026.1851820 (PMC13650467; doi:10.3389/fbioe.2026.1851820)
Supplement: Supplementary file 1 [file Supplementaryfile1.docx]

**Table 1.** b.Spine study: Fusion assessment

|  |  |  |  |  |  | **Fusion assessment  at 6 months by site investigator** | | **Fusion assessment  at 12 months by site investigator** | |
| --- | --- | --- | --- | --- | --- | --- | --- | --- | --- |
| **Patient** | **Age** | **Diagnosis** | **Number of total spinal levels involved** | **Spinal level** | **Treated with b.Bone** | **Left Side grade** | **Right Side grade** | **Left Side grade** | **Right Side grade** |
| **01-001** | 57 years | Degenerative disc disease | 8 | **L2-L3** | **Yes** | **3** | **4** | **4** | **5** |
|  |  |  |  | **L3-L4** | **Yes** | **3** | **4** | **5** | **5** |
|  |  |  |  | **T10-T11** | **No (autologous bone)** | **1** | **1** | **1** | **1** |
|  |  |  |  | **T11-T12** | **No (autologous bone)** | **1** | **1** | **2** | **2** |
|  |  |  |  | **L5-S1** | **No (TLIF)** | **Not assessable (carbon cage)** | **Not assessable (carbon cage)** | **Not assessable (carbon cage)** | **Not assessable (carbon cage)** |
| **01-002** | 74 years | Stenosis - Degenerative disc disease | 8 | **T10-T11** | **Yes** | **2** | **3** | **3** | **4** |
|  |  |  |  | **L3-L4** | **No (titanuim cage)** | **Not applicable** | **Not applicable** | **Not applicable** | **Not applicable** |
|  |  |  |  | **L4-L5** | **No (titanuim cage)** | **Not applicable** | **Not applicable** | **Not applicable** | **Not applicable** |
| **01-003** | 19 years | Deformity | 9 | **T4-T5** | **Yes** | **4** | **4** | **5** | **5** |
|  |  |  |  | **T5-T6** | **Yes** | **4** | **4** | **5** | **5** |
|  |  |  |  | **T7-T8** | **No** | **4** | **3** | **3** | **3** |
|  |  |  |  | **T8-T9** | **No** | **4** | **4** | **4** | **4** |
|  |  |  |  | **T9-T10** | **No** | **4** | **4** | **4** | **4** |
|  |  |  |  | **T10-T11** | **No** | **3** | **3** | **3** | **3** |
|  |  |  |  | **T11-T12** | **No** | **3** | **3** | **3** | **3** |
|  |  |  |  | **T12-L1** | **No** | **3** | **3** | **4** | **4** |
| **01-005** | 59 years | Deformity - Degenerative disc disease | 6 | **T4-T5** | **Yes** | **3** | **4** | **5** | **5** |
|  |  |  |  | **T5-T6** | **Yes** | **3** | **4** | **5** | **4** |
|  |  |  |  | **T6-T7*** | **Yes** | **3** | **3** | **4** | **3** |
|  |  |  |  | **T12-L1** | **No** | **3** | **3** | **4** | **4** |
|  |  |  |  | **L1-L2** | **No** | **3** | **3** | **4** | **4** |
| **01-006** | 33 years | Deformity | 8 | **T7-T8** | **Yes** | **4** | **4** | **5** | **5** |
|  |  |  |  | **T8-T9** | **Yes** | **5** | **5** | **5** | **5** |
|  |  |  |  | **T12-L1** | **No** | **Not assessable (bilateral laminectomy)** | **Not assessable (bilateral laminectomy)** | **Not assessable (bilateral laminectomy)** | **Not assessable (bilateral laminectomy)** |
|  |  |  |  | **L1-L2** | **No** | **Not assessable (bilateral laminectomy)** | **Not assessable (bilateral laminectomy)** | **Not assessable (bilateral laminectomy)** | **Not assessable (bilateral laminectomy)** |
|  |  |  |  | **L2-L3** | **No** | **Not assessable (dysmorphism)** | **Not assessable (dysmorphism)** | **Not assessable (dysmorphism)** | **Not assessable (dysmorphism)** |
| **01-007** | 58 years | Deformity | 8 | **T8-T9** | **Yes** | **3** | **3** | **4** | **4** |
|  |  |  |  | **T9-T10** | **Yes** | **3** | **3** | **4** | **4** |
|  |  |  |  | **T10-T11** | **No** | **4** | **3** | **4** | **4** |
|  |  |  |  | **T11-T12** | **No** | **3** | **3** | **4** | **3** |
|  |  |  |  | **T12-L1** | **No** | **2** | **2** | **3** | **4** |
|  |  |  |  | **L1-L2** | **No** | **4** | **Not assessable (arthrectomy)** | **4** | **Not assessable (arthrectomy)** |
|  |  |  |  | **L2-L3** | **No** | **4** | **Not assessable (arthrectomy)** | **4** | **Not assessable (arthrectomy)** |
|  |  |  |  | **L3-L4** | **No** | **4** | **4** | **5** | **5** |
| **01-008** | 57 years | Deformity | 8 | **T9-T10** | **Yes** | **3** | **3** | **4** | **5** |
|  |  |  |  | **T10-T11** | **Yes** | **3** | **4** | **4** | **5** |
|  |  |  |  | **T11-T12** | **Yes** | **3** | **4** | **4** | **5** |
|  |  |  |  | **T12-L1** | **No** | **2** | **2** | **3** | **3** |
|  |  |  |  | **L1-L2** | **No** | **2** | **2** | **2** | **3** |
|  |  |  |  | **L2-L3** | **No** | **2** | **3** | **3** | **4** |
|  |  |  |  | **L3-L4** | **No** | **3** | **5** | **4** | **5** |
|  |  |  |  | **L4-L5** | **No** | **3** | **Not assessable (cage)** | **4** | **Not assessable (cage)** |

T6-T7* - CT scan images show that only a small amount of b.Bone is present

**Successful fusion** is defined as “probable radiographic fusion - 4” or “Radiographic fusion - 5”

**Note:** According to the study protocol, up to three spinal levels could be treated with b.Bone, and consequently, the other levels were treated according to the standard of care without b.Bone.

**Table 2.** Instrumented Posterolateral Fusion with benchmark synthetic ceramic bone substitutes

| **Reference** | **Device** | **Sample Size**  **Analysed** | **Fusion**  **Procedure** | **Anatomic**  **al Region** | **Extender/**  **standalone** | **Performance** | **Fusion**  **Evaluation** | **Safety Summary** | **Evidence for**  **Benefit** | **Risk Evidence** |
| --- | --- | --- | --- | --- | --- | --- | --- | --- | --- | --- |
| **Lehr AM, et al.,** Efficacy of a Standalone Microporous Ceramic Versus Autograft in Instrumented Posterolateral Spinal Fusion: A Multicenter,  Randomized, | **AttraX Putty** Microporous Ceramic | 87  Intrapatient Controlled. | Instrumente d PLF, 62%  of which had additional interbody fusion. | Thoraco-lumbar | Standalone | Fusion rate at 12 months  55% for the AttraX® Putty side and 52% for the autograft side, with an overall fusion rate of 71%.  CT scans | Fusion: continuous bony bridge from one vertebra to the other, in the absence of any secondary signs of nonunion such as fracture or loosening of the screws and rods | 110 (serious) adverse events were registered in 48 patients. | Non-inferior fusion vs autograft alone in instrumented PLF. High-quality evidence supports ceramic equivalence. | No difference in complications vs autograft. |
| Intrapatient Controlled, Noninferiority Trial. *Spine (Phila Pa 1976), 2020. 45(14): p. 944-*  *951.* |  |  |  |  |  |  | screws or rods |  |  |  |
| **Kanter AS, et al.,** A prospective, multi-center clinical and radiographic outcomes evaluation of ChronOS strip for lumbar spine fusion. *J Clin Neurosci, 2016. 25: p. 36-40* | **ChronOS strip** | 76  (55 at 24  months) | Instrumente d PLF+  interbody spacer in 68.8% | Lumbar | Extender 1:1 | Fusion achieved in 88.9%  patients at 12months and in 91.8%  patients at 24months Xrays.  CT scan at 12 and 24 months when fusion was dubious | Composite endpoint for postero-lateral fusion success:  - bridging bone per Lenke scale possibly or definitely solid  -translational motion durig flex/ext (<3mm)  -Angular motion during flex/ext (<5mm)  -Posterior hardware status intact | 1 hardware failure, which did not affect treatment  and was unrelated to ChronOs strip. 3 secondary interventions: 1 wound infection, 1 nerve root impingement, and 1 cage migration | Acceptable fusion outcomes when combined with autograft. | No increased risk; similar complication rates. |
| **Epstein NE.** A preliminary study of the efficacy of beta tricalcium phosphate as a bone expander for instrumented posterolateral lumbar fusions. *J Spinal Disord Tech.* 2006;19(6):424–429. | **Vitoss** + lamina autograft | 40 (27 single-  level; 13 two-level) | Instrumente d posterolater al fusion | Lumbar | Extender 1:1 | **Single-level**: 26/27 fused (96.3%); **Two-**  **level:** 11/13 fused (84.6%); small number of pseudarthrose s (1 single-  level, 2 two-level). SF-36 + Odom criteria improved by 12 months. | Dynamic radiographs and 2D CT at 3, 6, 12  month | No major device-related complications; 1 revision for symptomatic pseudarthrosis | High fusion rates; good/excellent clinical outcomes | Slightly lower success for two-level; smoking risk factor |
| **Epstein NE.** Beta tricalcium phosphate: observation of use in  100 posterolateral | **Vitoss** + lamina autograft | 100 (79  single-level; 21 two-level) | Instrumente d Posterolater al fusion  after | Lumbar | Extender 1:1 | **Single-level**: 76/79 fused (96.2%). | Dynamic X-rays + CT | No major device-related complications; smoking correlated with | Excellent fusion rates; strong clinical improvement | Smoking and multi-level fusion increase risk of delay/  nonunion |
| lumbar instrumented fusions. *Spine J.*  2009;9(8):630–638. |  |  | multilevel laminectomi es |  |  | **Two-level**: 19/21 fused (90.5%).  By 12 months, fusion rates similar between groups. |  | delayed fusion. Single-level: 3 pseudarthroses. Two-level: 2 pseudarthroses. |  |  |
| **Lerner T., at al.** A level-1 pilot study to evaluate ultraporous β-tricalcium phosphate as a graft extender in the posterior correction of adolescent idiopathic scoliosis. *Eur Spine J, 2009; 18:170–179.* | **Vitoss +** local autologous (β-TCP  group)  vs ICBG +  local (ICBG group) | 40 (AIS) | Instrumente d Posterior correction and fusion | Thoraco-lumbar | Extender | β-TCP group (Vitoss): 19  out of 20 patients fused (95%). | X- rays Radiographs were examined for:    - Continuous and mature fusion mass    - Implant integrity    - loss of curve correction greater than 10° | One pseudarthrosis in β-TCP group; donor site pain in ICBG group | Equivalent fusion results to ICBG; avoids donor site morbidity.  Comparable to ICBG; curve correction: 61.7%  vs 61.2% post-op;  57.2% vs 54.3% at  48 months follow-up | No increased complications vs ICBG |
| **Stempels H W et al.,** Efficacy of Biphasic Calcium Phosphate Ceramic With a Needle-Shaped Surface Topography Versus Autograft in Instrumented Posterolateral Spinal Fusion. *Spine (Phila Pa 1976). 2024 Oct*  *1;49(19):1323-1331* | **MagnetOs** BCP needle-shaped  ceramic | 91  Intrapatient Controlled. | Instrumente d PLF with or without interbody devices | Thoraco-lumbar | Standalone | At 12 months follow-up  - 79%  MagnetOs side vs 47%  autograf side.  - Overall posterolateral fusion rate per segment (left and/or right): 83%  CT scan | According to Christensen (2001) and  Carreon (2007), the level was  considered  “fused” if there was bridging bone visible on the CT in the posterolateral  gutters. From the figures in the paper, they  looked for continuous radiodense (trabeculated) bone between transverse processes. | Acceptable; no increased device-related risks | Superior fusion vs autograft | No increased risks |
| **Zanotti B., et al**, Efficacy and safety of the magnesium-hydroxyapatite bone graft substitute in postero-lateral spinal fusion: observational, spontaneous clinical study. *Progress in Neuroscience 2021;*  *6 (1-4): 27-35.* | **SintLife** Magnesium-doped hydroxyapati te | 20 | Interbody  Instrumente d postero-lateral fusion | Thoracic | Standalone (no autograft control) | 73.4% at 12  months CT scans | Brantigan Steffe classification | One minor adverse event (superficial inflammation), resolved without surgical revision. Significant improvement in ODI and VAS scores; fusion rate comparable to literature for HA-based grafts | Minimal risk: one minor inflammation, no major complications |  |
| **Giorgi P., et al,** Use of a novel porous hydroxyapatite bone graft substitute in postero-lateral and interbody spinal fusion: clinical and radiographic analysis at 4 year follow up. *Progress in Neuroscience 2015;*  *3 (1-4): 117-124.* | **Engipore** | 36 (19 in  posterolater al group and 17 in interbody group) | Two groups. Group A: Instrumente d posterolater al fusion Group B: interbody fusion (with transforamin al approach) | Thoraco-lumbar | Extender 2:1 | About 90% at  12 months (confirmed at 4 years: 89.4%  PLF, 94.1%  interbody) Xrays or CTscans | Lenke classification for postero-lateral fusion  Brantigan Steffe classification for interbody fusion | No major complications; no scaffold-related adverse events | Significant improvement in ODI and VAS; high fusion rates comparable to autologous bone | Minimal risk: one screw mobilization, no infection or graft-related issues. |
| **Griffoni G., et al.,** Ceramic bone graft substitute (Mg-HA) in spinal fusion: A prospective pilot study. *Front. Bioeng. Biotechnol., 17*  *November 2022 Sec. Biomaterials,*  *Volume 10 – 2022.* | **SintLife** Magnesium-doped hydroxyapati te | 16 | Instrumente d Posterolater al Fusion + Interbody Fusion (TLIF) in 13/16 | Lumbar | Standalone | About 62% at  12 months CT scans | Brantigan Steffe classification, which assesses the spinal fusion from grade A (pseudoarthrosis) to grade E (certain fusion). | No device-related adverse events; 3 inflammatory reactions (not related to device) | Significant improvement in ODI, VAS, EQ-5D;  confirms osteointegration and remodeling | Minimal risk: inflammatory events linked to patient conditions, not device |
